# Sweat output measurement of the post-ganglion sudomotor response by Q-Sweat Test: a normative database of Chinese individuals

**DOI:** 10.1186/1471-2202-13-62

**Published:** 2012-06-08

**Authors:** Shu-Fang Chen, Ya-Ting Chang, Cheng-Hsien Lu, Chi-Ren Huang, Nei-Wen Tsai, Chiung-Chih Chang, Chih-Cheng Huang, Yao-Chung Chuang, Wen-Neng Chang

**Affiliations:** 1 Department of Neurology, Kaohsiung Chang Gung Memorial Hospital and Chang Gung University College of Medicine, Kaohsiung, Taiwan; 2 Department of Biological Science, National Sun Yat-Sen University, Kaohsiung, Taiwan

**Keywords:** Age, Body height, Body mass index, Body weight, Chinese, Gender, Normative database, Q-Sweat, Sweat output

## Abstract

****Background**:**

Q-Sweat is a model used for evaluating the post-ganglionic sudomotor function by assessing sweat response. This study aimed to establish the normative database of Q-Sweat test among Chinese individuals since this type of information is currently lacking.

****Results**:**

One hundred and fifty (150) healthy volunteers, 76 men and 74 women with age range of 22–76 years were included. Skin temperature and sweat onset latency measured at the four sites (i.e., the forearm, proximal leg, distal leg, and the foot) did not significantly correlate with age, gender, body height (BH), body weight (BW), and body mass index (BMI) but the total sweat volume measured in all four sites significantly correlated with sex, BH, and BW. Except for the distal leg, the total sweat volume measured at the other three sites had a significant correlation with BMI. In terms of gender, men had larger total sweat volume, with median differences at the forearm, proximal leg, distal leg, and foot of 0.591 μl, 0.693 μl, 0.696 μl, and 0.358 μl, respectively. Regarding BW difference (≥62 and < 62 Kg), those with BW ≥62 Kg had larger total sweat volume. Median differences at the forearm, proximal leg, distal leg, and foot were 0.538 μl, 0.744 μl, 0.695 μl, and 0.338 μl, respectively. There was an uneven distribution of male and female participants in the two BW groups. In all conditions, the total sweat volume recorded at the foot site was the smallest.

****Conclusion**:**

This is the first report to show the normative database of sweat response in Chinese participants evaluated using Q-Sweat device. This normative database can help guide further research on post-ganglionic sudomotor or related clinical practice involving a Chinese population.

## **Background**

Autonomic nervous system (ANS) dysfunction may manifest in a variety of symptoms, including changes in sweating, either in an increased or decreased manner. Currently, sweat production, a sudomotor response of ANS, can be measured using several tools such as thermoregulatory sweat testing (TST), quantitative sudomotor axon reflex testing (QSART), silicone impressions, sympathetic skin response (SSR), acetylcholine (Ach) sweat-spot test, and quantitative direct and indirect axon reflex testing (QDIRT) [1,2]. The QSART, designed by Low et al. [3], is used clinically to evaluate post-ganglionic sudomotor function by measuring the axonal reflex-mediated sweat response over time, with the stimulation of sweat glands by 10% Ach. It is sensitive and reproducible in both controls and subjects with neuropathies [3-7]. The Q-Sweat is a commercial quantitative sweat measurement system which examines the indirect sweat response, is modeled on QSART [8]. A study conducted by Sletten et al. [8] to investigate the sweat response using Q-Sweat and QSART recording under identical conditions in healthy normal controls shows significantly lower volumes in each of the four measured sites. This comparative result can be used to estimate the expected QSART volume given an observed Q-Sweat volume, although it is preferable to use the Q-Sweat normative database directly. So far, the normal databases of the QSART and Q-Sweat are often limited to Caucasians [7,8] and there is a lack of a normative database for Asians. The aim of this study was to establish the normal databases of sudomotor function among the Chinese using the Q-Sweat device, and to examine the factors that may influence the total volume of sweat response.

## **Methods**

### **Participants**

After approval of the Ethics Committee of Chang Gung Memorial Hospital (IRB 98-0805B), 150 healthy volunteers (76 men, 74 women) aged 22–76 years were examined by a neurologist before participating in the study. All were free of systemic diseases that might affect the ANS and were not taking any medicine that might affect autonomic functions (e.g., aspirin, alpha-blocker, beta-blocker, calcium channel blocker, cholinergic or anti-cholinergic agents, and anti-acetylcholinesterase and serotonergic agents). Tea and coffee consumption, and smoking were also not allowed at least one day prior to the test. None of the participants had any dermatologic illnesses including skin injuries, or was a substance abuser. Each participant provided written informed consent. All of them were examined in the morning time, and their age, gender, body weight (BW), body height (BH) and body mass index (BMI) were checked on the day of the study.

### **Procedures and recordings**

The Q-Sweat studies were performed under controlled room temperature and humidity with a Q-sweat device (WR Medical Elextronics Co., Stillwater, Minnesota, USA). During the examination, the participant was placed in a supine position and underwent Q-Sweat recordings on the left side of body only because it was assumed that there would be no difference in ANS evaluation between the right and left sides [9].

**Figure 1:**
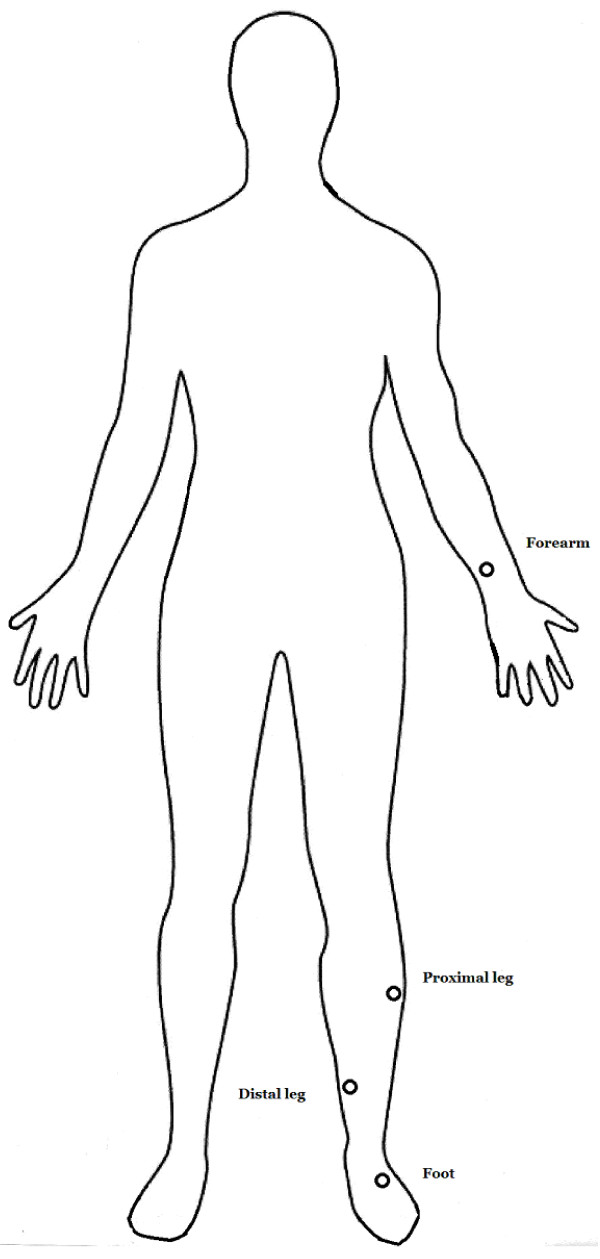
The four recording sites were the forearm (medial forearm site three-fourths of the distance from the ulnar epicondyle to the pisiform bone), proximal leg (5 cm distal to the fibular head), distal leg (5 cm proximal to the medial malleolus), and the foot (over the extensor digitorum brevis).

In the study, four skin regions, as suggested [1,9-11] were recorded in a fixed manner (Figure 1). These were the skin surface of the medial forearm (innervated by the ulnar nerve), the proximal leg (innervated by the peroneal nerve), the distal leg (innervated by the saphenous nerve), and the proximal foot over the extensor digitorum brevis (innervated by the sural nerve). Before the study, the four skin regions were cleaned with soap and water, and thoroughly dried with absorbent paper and shaved if necessary. The regional limb skin temperature was recorded by infrared thermometer. If it was <30.0°C, the examined limbs were warmed-up with ultra-red heat lamp, but not >33.5°C in order to ensure adequate blood flow while remaining below the limb sweating threshold [4]. The agent used for evoking the sweat response was 10% w/v Ach solution, which was applied to fill the chamber of the Meridian electrode (PN#5191, WR Medical Electronics Co.) that was affixed to the skin at the test site.

The full description of the Q-Sweat device can be obtained from the manufacturer (WR Medical Electronics Co.). This study followed the recording procedure described in the report of Sletten et al. [8]. Briefly, the Q-Sweat device used a desiccant pack (#5190; WR Medical Electronics Co., Stillwater, Minnesota, USA) as its dry air source. Room air was drawn in through an intake pump and channeled through a serpentine of drierite (W. A. Hammond Co., Xenia, Ohio). This air was then passed through a set of sensors (Honeywell International, Inc., Morristown, New Jersey) that controlled the flow rate. The sensors evaluated the temperature and percentage of relative humidity. Lastly, the dried air was delivered to the capsule assembly and applied to the regional skin. Moisture released from the human eccrine sweat gland was picked up by the dried room air and returned to the main unit of the device via Teflon-lined Tygon tubing. The sensors again evaluated the temperature and percent relative humidity along with flow rate. These values were compared to the baseline (initial) values and integrated using the vapor pressure calculation for water between 0°C and 50°C.

Sweat rate, expressed as nano-liters per minute was then displayed using the TestWorks software (WR Medical Electronics Co., Stillwater, Minnesota, USA). A constant-current stimulator, the Iontophor II (Model 6111 PM/DX, Life Tech, Inc. Stafford, Texas), was used in conjunction with the Q-Sweat device. Sweat amount and onset time of response were displayed using TestWorks software. Both sweat latency and volume were recorded. The latency measurement was the “on” time of the noticeable sweat rate change after the stimulation, and was displayed as minutes. The volume measurement was the sweat response from the time of onset latency to the end of 5 min stimulation and another 5 min recording (total of 10 min recording), and was displayed as micro-liters per 10 min.

### **Statistical analysis**

For statistical analysis, the Statistical Package for Social Sciences (SPSS) software package (version 13 for Windows®, SPSS Inc, Chicago, IL) was used. All of the data were displayed as median (minimum, maximum). Differences in basic demographic data (i.e., BH, BW, BMI, room temperature, and room humidity) between genders was calculated by the Mann–Whitney *U* test and together with skin temperature, onset latency and total volume of sweat response, were check by the Spearman correlation analysis. A two-tailed *p* < 0.01 was considered significant.

Before entering the linear regression, the sweat volumes were tested for normality by the Kolmogorov-Smirnov test and were calculated by square root to transform them into normal distribution. Linear regression was introduced to check the significant varieties revealed by the Spearman correlation in a “Stepwise Method” for all of the varieties. The equations, R and R-squared were calculated. Differences in skin temperature, onset latency and volume at each of the four sites by gender and by BW (cut- off point 62 Kg) were checked by the Mann–Whitney *U* test, while the Kruskal-Wallis Test was used to check the differences in five age ranges (i.e., 20–30, 30–40, 40–50, 50–60 and ≥61 years). A *p* < 0.05 was considered significant.

## **Results**

The demographic data of the 150 healthy participants showed significant differences in BH, BW, and BMI between sexes (Table 1). Results of the respective correlation analyses between the sweat output responses and age, gender, BH, BW, and BMI are listed in Table 2. Skin temperatures and sweat onset latencies at the four measured sites did not significantly correlate with age, sex, BH, BW, and BMI. However, the total volume of sweat response measured on all four sites significantly correlated to gender, BH, BMI and BW. After multiple linear regression analysis, BW was the factor that influenced the sweat volume measured on all four sites and was the only factor that positively correlated with the total volume of sweat response measured at the forearm, and proximal and distal leg. Thus, the higher BW meant larger sweat volume (Tables 3 and 4). Between participants with BW ≥62 Kg and those with BW <62 Kg, gender, BH and BMI were significantly different (Table 5).

**Table 1:** Demographic data of the participants (n = 150)

	**Total**	**Woman**	**Man**	**Sig.**
**Number**	**150**	**74**	**76**	
Age	44.0 (22.0, 76.0)	44.5 (22.0, 76.0)	43.0 (24.0, 73.0)	0.758
Body Height (m)	1.64 (1.45, 1.84)	1.56 (1.45, 1.68)	1.71 (1.53, 1.84)	0.000*
Body Weight (Kg)	61.0 (45.0, 95.0)	55.0 (45.0, 87.8)	69.5 (53.0, 95.0)	0.000*
Body mass index	22.7 (17.1, 35.6)	21.6 (17.1, 35.6)	23.5 (17.9, 34.9)	0.000*
Room wet (%)	56.0 (49.0, 61.0)	56.0 (49.0, 60.0)	56.0 (54.0, 61.0)	0.593
Room temperature (°C)	23.0 (19.0, 27.0)	23.0 (20.0, 27.0)	23.0 (19.0, 26.0)	0.800

Data presented as median (maximum, minimum).

Abbreviations: m, meter; Kg, kilogram; Sig, significance (tested by Mann–Whitney *U* test).

*Significant correlation at the 0.05 level (2-tailed).

**Table 2:** Results of correlation analysis between the sweat output response and demographic data

**Spearman’s rho**	**Gender**		**Age**		**Body Height**		**Body Weight**		**Body Mass Index**	
**Correlations**	**Cor. Coe.**	**Sig.**	**Cor. Coe.**	**Sig.**	**Cor. Coe.**	**Sig.**	**Cor. Coe.**	**Sig.**	**Cor. Coe.**	**Sig.**
Skin temperature (°C)										
Forearm	0.057	0.486	0.066	0.422	0.021	0.796	−0.049	0.796	−0.098	0.231
Proximal leg	0.182	0.026	0.190	0.020	0.109	0.183	−0.033	0.686	−0.033	0.686
Distal leg	0.132	0.107	0.145	0.077	0.079	0.339	0.027	0.742	−0.056	0.496
Foot	−0.012	0.885	−0.007	0.934	−0.054	0.515	0.120	0.144	0.180	0.028
Latency (min)										
Forearm	−0.044	0.593	−0.140	0.087	−0.018	0.829	−0.115	0.162	−0.173	0.034
Proximal leg	−0.145	0.077	−0.055	0.502	−0.092	0.263	−0.103	0.210	−0.091	0.269
Distal leg	−0.143	0.081	−0.165	0.044	−0.119	0.146	−0.101	0.219	0.035	0.671
Foot	0.007	0.928	0.077	0.150	−0.096	0.244	−0.067	0.416	−0.010	0.905
Volume (μl)										
Forearm	0.366	0.000*	0.170	0.037	0.287	0.000*	0.447	0.000*	0.357	0.000*
Proximal leg	0.448	0.000*	−0.064	0.436	0.397	0.000*	0.466	0.000*	0.282	0.000*
Distal leg	0.496	0.000*	−0.139	0.090	0.467	0.000*	0.478	0.000*	0.219	0.007*
Foot	0.455	0.000*	−0.218	0.007*	0.441	0.000*	0.514	0.000*	0.309	0.000*

Abbreviations: Sig, significance (tested by Spearman’s rho correlation analysis); Cor Coe, Correlation coefficient.

*Correlation was significant at the 0.01 level (2-tailed).

**Table 3:** Multiple linear regression “stepwise” analysis for total volumes of sweat response of the four measured sites with sex, age, BH, BW and BMI

**Sweat volume (μl)**	**Forearm (Vt1)**	**Proximal leg (Vt2)**	**Distal leg (Vt3)**	**Foot (Vt4)**
**(R, R square)**	**(weight = 0.465, 0.216)**	**(weight = 0.436, 0.190)**	**(weight = 0.452, 0.204)**	**(weight; age; sex = 0.556, 0.310)**
Non-standardized regression equation	Vt1 = −0.216 + 0.017 × BW	Vt2 = 0.038 + 0.014× BW	Vt3 = −0.175 + 0.017 × BW	Vt4 = 0.213 + 0.007 × BW - 0.003 × age + 0.119× gender
Standardized regression equation	Vt1 = 0.465 × BW	Vt2 = 0.436 × BW	Vt3 = 0.452 × BW	Vt4 = 0.319 × BW - 0.186 × age + 0.246× gender

Abbreviations: BH, body height; BW, body weight; BMI, body mass index; Vt1, square root of total volume of sweat response recorded at forearm; Vt2, square root of total volume of sweat response recorded at proximal leg; Vt3, square root of total volume of sweat response recorded at distal leg; Vt4, square root of total volume of sweat response recorded at foot.

**Table 4:** Linear regression “enter” analysis for the total volumes of sweat response of the four measured sites with age

**Sweat volume (μl)**	**Forearm (Vt1)**	**Proximal leg (Vt2)**	**Distal leg (Vt3)**	**Foot (Vt4)**
**(R, R square)**	**(0.173, 0.030)**	**(0.057, 0.003)**	**(0.119, 0.014)**	**(0.203, 0.041)**
Non-standardized regression equation	Vt1 = 0.640 + 0.005 × age	Vt2 = 1.007 - 0.002× age	Vt3 = 1.022 – 0.003 × age	Vt4 = 0.739 - 0.004 × age
Standardized regression equation	Vt1 = 0.173 × age	Vt2 = −0.057 × age	Vt3 = −0.119 × age	Vt4 = − 0.203 × age

Abbreviations: Vt1, square root of total volume of sweat response recorded at forearm; Vt2, square root of total volume of sweat response recorded at proximal leg; Vt3, square root of total volume of sweat response recorded at distal leg; Vt4, square root of total volume of sweat response recorded at foot.

**Table 5:** Basic information of the participants by body weight ≥62 and <62 Kg

**Body weight (Kg)**	**Total**	**≥62**	**<62**	**Sig.**
**Case number**	**150**	**74**	**76**	
**Gender (male: female)**	**76:74**	**64:10**	**12:64**	0.000*
Age (y/r)	44.0 (22.0, 76.0)	44.0 (22.0, 73.0)	44.0 (23.0, 76.0)	0.759
Body height (m)	1.64 (1.45, 1.84)	1.70 (1.48, 1.84)	1.58 (1.45, 1.77)	0.000*
Body mass index	22.7 (17.1, 35.6)	24.2 (18.9, 35.6)	21.0 (17.1, 27.8)	0.000*
Room wet (%)	56.0 (49.0, 61.0)	55.5 (49.0, 61.0)	56.0 (50.0, 61.0)	0.254
Room temperature (°C)	23.0 (19.0, 27.0)	23.0 (19.0, 26.0)	23.0 (20.0, 27.0)	0.577

Data presented as median (maximum, minimum).

Abbreviations: Kg, kilogram; m, meter; Sig, significance as tested by the Mann–Whitney *U* test.

*Significant correlation at the 0.05 level (2-tailed).

**Figure 2:**
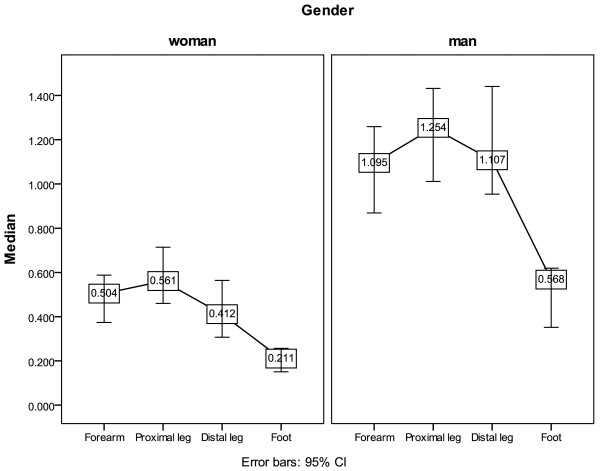
**Difference in total volume of sweat response by gender (unit: μl).** The median sweat output volume in males was larger than that of females. CI, confidence interval.

**Figure 3:**
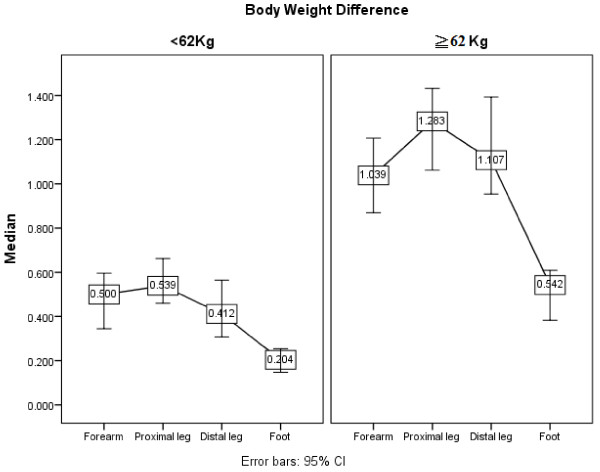
**Difference in total volume of sweat response by body weight difference (≥62 and <62 kilograms [kg]) (unit: μl). **The median sweat output volume of the participants with body weight ≥62 kg was larger than that of participants with <62 kg body weight. CI, confidence interval.

The sweat responses presented as skin temperature, onset latency, and total volume of sweat response by gender and BW (≥62 Kg and <62 Kg) are shown in Tables 6 and 7, respectively. Gender difference did not significantly influence skin temperature and onset latency of sweat response, but had a significant difference on the total volume of sweat response in all the four recorded regions (Table 6; Figure 2). The median differences at the forearm, proximal leg, distal leg and foot were 0.591 μl, 0.693 μl, 0.696 μl, and 0.358 μl, respectively. The BW difference did not have significant influence on skin temperature and onset latency of sweat response, but had a significant influence on the total volume of sweat response in all four recorded regions (Table 7; Figure 3). The median differences at the forearm, proximal leg, distal leg and foot were 0.583 μl, 0.744 μl, 0.695 μl and 0.338 μl, respectively. Tables 6 and 7 also show that the total volume of sweat response recorded at the foot was the smallest compared to those recorded at other sites (Tables 6 and 7). In terms of sweat response in different age groups by Kruskal-Wallis Test, there was no statistical difference between the five groups (Table 8).

**Table 6:** Sweat output detected by Q-sweat and skin temperature over the four skin regions by gender

	**Total**	**Male**	**Female**	**Sig.**
**Number**	**150**	**76**	**74**	
Skin temperature (°C)				
Forearm	35.8 (34.0, 37.7)	35.9 (34.1, 37.7)	35.8 (34.0, 37.3)	0.484
Proximal leg	36.2 (34.2, 38.0)	36.3 (34.2, 37.7)	36.0 (34.5, 38.0)	0.126
Distal leg	35.6 (34.0, 37.2)	35.7 (34.0, 37.0)	35.4 (34.0, 37.2)	0.107
Foot	35.8 (34.0, 37.3)	35.8 (34.0, 37.3)	35.7 (34.0, 37.2)	0.885
Onset latency (minute)				
Forearm	2.2 (0.6, 3.9)	2.1 (1.0, 3.9)	2.2 (0.6, 3.5)	0.591
Proximal leg	1.9 (0.7, 4.0)	1.8 (0.7, 2.9)	2.0 (0.7, 4.0)	0.077
Distal leg	1.8 (0.6, 3.8)	1.7 (0.7, 3.5)	2.0 (0.6, 3.8)	0.081
Foot	2.3 (0.8, 4.4)	2.3 (0.8, 3.8)	2.2 (0.9, 4.4)	0.928
Total sweat volume (μl)				
Forearm	0.748 (0.02, 3.983)	1.095 (0.023, 3.983)	0.504 (0.020, 3.814)	0.000*
Proximal leg	0.825 (0.029, 4.493)	1.254 (0.029, 3.753)	0.561 (0.060, 4.493)	0.000*
Distal leg	0.687 (0.015, 4.914)	1.107 (0.043, 4.821)	0.411 (0.015, 4.914)	0.000*
Foot	0.294 (0.014, 1.718)	0.568 (0.041, 1.718)	0.210 (0.014, 0.802)	0.000*

Data presented as median (maximum, minimum).

Abbreviations: Sig, significance tested by the Mann–Whitney *U* test.

*Significant correlation at the 0.05 level (2-tailed).

**Table 7:** Sweat output detected by Q-sweat and skin temperature over four skin regions by BW ≥62 and <62 Kg

**Weight (Kg)**	**Total**	**≥62**	**<62**	**Sig.**
**Number**	**90**	**74**	**76**	
Skin temperature (°C)				
Forearm	35.8 (34.0, 37.7)	35.8 (34.0, 37.7)	35.8 (34.1, 37.3)	0.648
Proximal leg	36.2 (34.2, 38.0)	36.3 (34.2, 37.2)	36.1 (34.6, 38.0)	0.981
Distal leg	35.6 (34.0, 37.2)	35.7 (34.0, 37.0)	35.5 (34.0, 37.2)	0.821
Foot	35.8 (34.0, 37.3)	35.9 (34.0, 37.3)	35.6 (34.0, 37.2)	0.517
Onset latency (minute)				
Forearm	2.2 (0.6, 3.9)	2.1 (1.0, 3.9)	2.2 (0.6, 3.8)	0.450
Proximal leg	1.9 (0.7, 4.0)	1.8 (0.7, 2.9)	1.9 (0.7, 4.0)	0.780
Distal leg	1.8 (0.6, 3.8)	1.7 (0.6, 3.5)	2.0 (0.6, 3.8)	0.588
Foot	2.3 (0.8, 4.4)	2.3 (0.8, 3.8)	2.3 (0.9, 4.4)	0.762
Total sweat volume (μl)				
Forearm	0.748 (0.020, 3.983)	1.038 (0.023, 3.983)	0.500 (0.020, 1.872)	0.000*
Proximal leg	0.825 (0.029, 4.493)	1.282 (0.029, 4.493)	0.538 (0.060, 2.821)	0.000*
Distal leg	0.687 (0.015, 4.914)	1.107 (0.043, 4.914)	0.412 (0.015, 2.902)	0.000*
Foot	0.294 (0.014, 1.718)	0.542 (0.032, 1.718)	0.204 (0.014, 0.952)	0.000*

Data presented as median (maximum, minimum).

Abbreviations: Kg, kilograms; BW, body weight; Sig, significance tested by the Mann–Whitney *U* test.

*Significant correlation at the 0.05 level (2-tailed).

**Table 8:** Sweat output responses detected by Q-Sweat device of the five different age groups

**Age Range**	**21-30**	**31-40**	**41-50**	**51-60**	**≥ 61**	**Sig.**
**Number**	**30**	**36**	**31**	**32**	**21**	**K-W**
Gender						
(men: women)	15:15	20:16	16:15	15:17	10:11	0.961
Height (m)	1.69 (1.49, 1.83)	1.66 (1.52, 1.83)	1.65 (1.53, 1.84)	1.61 (1.45, 1.80)	1.57 (1.48, 1.72)	0.003*
Weight (Kg)	63.0 (46.0, 85.0)	61.0 (46.0, 95.0)	60.0 (45.0, 89.0)	62.0 (47.0, 87.8)	61.2 (47.0, 89.0)	0.919
Body Mass Index	21.8 (17.9, 26.8)	21.9 (18.1, 34.9)	22.0 (17.1, 31.5)	23.3 (19.3, 35.6)	23.1 (18.6, 31.5)	0.041*
Room Wet (%)	56.0 (51.0, 61.0)	56.0 (50.0, 60.0)	56.0 (53.0, 61.0)	56.0 (49.0, 60.0)	56.0 (54.0, 60.0)	0.824
RT (°C)	23.0 (21.0, 25.0)	22.0 (19.0, 27.0)	22.0 (20.0, 26.0)	22.0 (21.0, 26.0)	22.0 (21.0, 24.0)	0.560
Skin temperature (°C)						
Forearm	35.8 (34.1, 37.0)	35.9 (34.1, 37.3)	35.8 (34.3, 37.3)	35.8 (34.0, 37.0)	36.4 (34.5, 37.7)	0.620
Proximal leg	36.0 (34.9, 36.7)	35.9 (34.6, 37.6)	36.1 (34.2, 37.7)	36.0 (34.5, 36.9)	36.0 (35.6, 38.0)	0.964
Distal leg	35.4 (34.0, 36.6)	35.4 (34.1, 36.9)	35.6 (34.1, 36.8)	35.7 (34.3, 37.2)	35.6 (34.0, 37.0)	0.635
Foot	36.0 (34.3, 36.8)	35.4 (34.0, 37.2)	35.7 (34.0, 37.2)	35.7 (34.1, 36.9)	36.0 (34.0, 37.3)	0.241
Onset latency (minute)						
Forearm	2.2 (0.8, 3.9)	2.3 (1.7, 3.8)	2.1 (0.8, 3.5)	2.1 (0.6, 3.1)	2.1 (1.3, 3.1)	0.164
Proximal leg	1.9 (0.7, 3.4)	1.9 (1.0, 4.0)	1.9 (0.7, 3.2)	1.8 (1.0, 2.6)	2.0 (1.0, 2.8)	0.945
Distal leg	2.2 (1.0, 3.2)	2.0 (0.7, 3.8)	1.7 (0.8, 3.0)	1.6 (0.6, 3.5)	1.9 (0.6, 2.8)	0.088
Foot	2.2 (1.0, 3.8)	2.3 (0.8, 3.6)	2.1 (1.0, 3.3)	2.4 (0.9, 4.4)	2.5 (1.3, 3.6)	0.099
Total Volume (μl)						
Forearm	0.594 (0.029, 3.983)	0.590 (0.020, 2.810)	0.873 (0.044, 3.018)	0.872 (0.128, 3.919)	0.887 (0.191, 2.416)	0.366
Proximal leg	1.068 (0.071, 2.755)	0.781 (0.029, 2.607)	0.657 (0.090, 2.821)	0.981 (0.108, 4.493)	0.662 (0.215, 1.697)	0.545
Distal leg	0.814 (0.295, 2.366)	0.635 (0.015, 2.902)	0.648 (0.107, 2.711)	0.763 (0.047, 4.914)	0.398 (0.084, 1.895)	0.322
Foot	0.376 (0.114, 1.095)	0.302 (0.014, 1.456)	0.296 (0.032, 1.718)	0.284 (0.036, 1.281)	0.212 (0.045, 0.609)	0.152

Data presented as median (maximum, minimum).

Abbreviations: K-W, Kruskal-Wallis Test; RT, Room Temperature; Sig, significance tested by the Kruskal-Wallis Test.

*Significant correlation at the 0.05 level (2-tailed.

## **Discussion**

For a PubMed search using the term “Q-Sweat”, only two related articles [8,10] can be found. Although QSART is an important tool for ANS evaluation, its clinical utility is still limited by its requisite of specialized equipment and its cost [1,2]. Compared with QSART, the Q-Sweat has a much simpler physiologic set-up. In the meanwhile, the Q-Sweat is reliable, reproducible, and easier to use, operate and maintain. Its study results can be used to estimate the expected QSART findings [8]. However, despite its importance in post-ganglionic sudomotor function evaluation, its clinical use is still limited, at least partially, due to the lack of normal values for reference, particularly among Asians, including the Chinese, who lack a normative database.

As shown in Tables 6,7 and 8, the onset latencies of sweat response are around 2.0 min, which is similar to that reported by Sletten et al. [8]. This may suggest that there is no ethnicity difference in sweat onset time. In the present study, the total volume of sweat responses was generally smaller than that reported by Sletten et al. [8]. It is known that different environmental conditions of study may contribute to the different sweat responses. The present study environment has a larger relative humidity (56% [49%, 61%]) than that of Sletten et al. (25-35%) (Tables 5 and 8) [8]. The room temperature of this study environment is 23°C (19°C, 27°C) compared to 23°C of Sletten et al. [8]. The influence of relative humidity on physiologic condition has also been reported recently by Maughan et al. [12], who found a similar sweat loss of 60% but higher sweat rate of 80% in relative humidity. In the meanwhile, the mean skin temperature was higher in a relative humidity at 80%. Thus, the physiologic responses may explain the relatively higher skin temperature of 35.5-36.0°C in the present study compared to the 31.1-32.6°C of Sletten et al. [8]. Therefore, the difference in the total volume of sweat response may imply that there is an ethnicity difference in sweat response.

There are studies [13-18] that examine racial or ethnic differences of ANS in different study methods. Although there may be differences in the number of sweat glands among different racial groups, other factors such as acclimatization may also influence the onset and type of sweating processes [15]. In the study of Johnson et al. [13], there was no significant difference in number of active sweat glands between black and white male subjects. The possible ethnicity difference in sweat response in the Q-Sweat test warrants further large-scale comparison study for better delineation. Nonetheless, the present study shows that the total volume of sweat response recorded at the foot is consistently the smallest when compared to those recorded at the forearm, proximal leg, and distal leg. This finding is also consistent with that reported by Sletten et al. [8] and is important for a relative comparison in an individual if no normal database is available for reference.

Gender difference is an important factor that may influence the total volume of sweat response (Tables 2 and 6), and this effect on sweat response is also noted in other reports [7,8,19]. In the present study (Table 6), the total volume of sweat response in males is about 2.0 times larger than that of females. This difference may be explained by the larger eccrine sweat gland droplets in men despite the same sweat gland density in both sexes [20].

The BW effect on ANS has been reported before [21] but all of the reported studies focus on cardiovascular responses that show a hyper-active sympathetic response and a hypo-active parasympathetic response. This change of ANS activity can also be observed in weight changes (weight gain or weight loss) [22-24]. The effect of BW on the sweat response has not been previously reported but in the present study, the BW is another factor that may influence the total volume of sweat response. As shown in Table 7, the total volume of sweat response of the participants with BW ≥62 Kg is about 2.0 times larger than those of the participants with a BW <62 Kg. However, there is an uneven distribution of male participants in the body weight groups, with a high percentage (86%, 64/74) in BW ≥62 Kg group and low percentage (16%,12/76) in BW <62 Kg (Table 7). This uneven distribution of gender percentage in these two different BW groups may have an influence on the sweat response. Further large scale and more even gender distribution studies are needed to establish a better delineation of the BW influence on sweat response.

The total volume of sweat response has a significant negative regression with age only in the foot site recording (Table 3). Based on analysis of the five separated age groups, aging causes lower total volume of sweat response in the foot site recordings (Table 8). Although this finding did not reach statistical significance, this effect of age on total volume of sweat response is also noted in others studies [7,19] whereby there is a progressive decline in the total volume of sweat response with age in all three lower extremity sites but not the forearm sites. In Table 4, the influence of age may have a similar effect on sweat response, but this difference cannot be drawn from the analysis shown in Table 8 because of the limited participants aged >60 years. In the report of Low et al. [19], aging has been shown to have selective influence on ANS activities. Of these, cardio-vagal function is known to be influenced significantly but not sweat response [3]. But as shown in the reports of Holowatt et al. [25] and Kihara et al. [26], aging may influence the sweat function.

The present study has limitations. First, the case number in the aged participants with complete normal ANS is limited. Second, there is an uneven distribution and gender and BW difference in the study groups. Third, the difference in laterality or sidedness is not examined. Lastly, participants of other ethnicities have not been included for comparison. Further large-scale study is needed to examine the sweat response of different groups of participants.

## **Conclusions**

Using the Q-Sweat study to evaluate the post-ganglionic sudomotor function by measuring sweat response, this study reveals the effect of gender on sweat response. The BW effect on sweat response can be influenced indirectly by the gender effect. This study also demonstrates that the total volume of sweat response recorded at the foot is consistently the smallest compared to those recorded at the forearm, proximal leg, and distal leg. This is the first report to show a normative database of sweat response evaluated using Q-Sweat devices among the Chinese. This normative database can be used for further post-ganglionic sudomotor research or clinical practice involving a Chinese population.

## **Competing interests**

All of the authors declare no competing or conflicts of interests.

## **Authors’ contributions**

All authors have read and approved the submitted manuscript. CSF and CYT contributed to the conception and design, data acquisition and analysis, and drafting and revision of the manuscript; LCH, HCR, TNW, CCC, and HCC contributed to the conception and design, and clinical data analysis; and CYC and CWN contributed to the conception and design, data analysis, and critical revision and final approval of the manuscript.
